# Supplementary material for: Assessing the Biofilm Formation Capacity of the Wine Spoilage Yeast Brettanomyces bruxellensis through FTIR Spectroscopy
Source: Microorganisms. 2021 Mar 12;9(3):587. doi: 10.3390/microorganisms9030587 (PMC7999561; doi:10.3390/microorganisms9030587)
Supplement: Supplementary file 1 [file microorganisms-09-00587-s001.pdf]

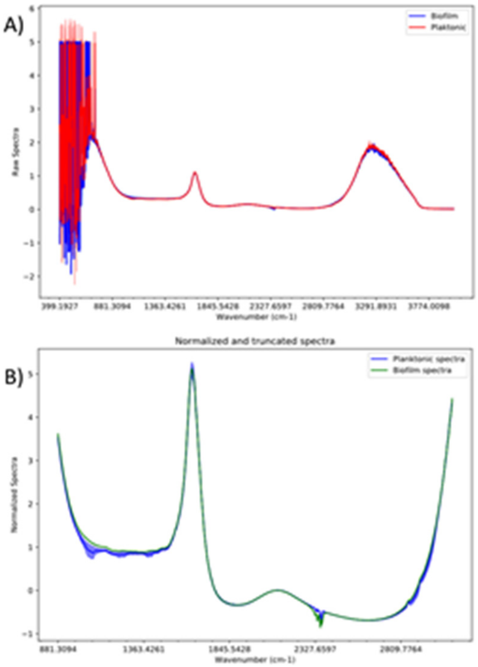

Figure S1. FTIR spectra: (**A**) Raw, unprocessed spectra; red line shows the mean planktonic spectrum, while the red shaded area shows the corresponding standard deviation at each wavenumber across all planktonic samples, the same holds true for the biofilm samples in blue; (**B**) Truncated at 880–3100 cm-1 and RNV normalized spectra, means and corresponding standard deviations; blue = planktonic samples and green = biofilm samples.
